# Molecular signatures bidirectionally link myocardial infarction and lung cancer

**DOI:** 10.3389/fmed.2025.1576375

**Published:** 2025-04-09

**Authors:** Dhruva Nandi, Rajiv Janardhanan, Sridhar Hannenhalli, Piyush Agrawal

**Affiliations:** ^1^Division of Medical Research, SRM Medical College Hospital & Research Centre, SRMIST, Kattankulathur, Chennai, Tamil Nadu, India; ^2^Cancer Data Science Laboratory, NCI, NIH, Bethesda, IL, United States

**Keywords:** myocardial infarction, lung cancer, survival analysis, machine learning, drug repurposing

## Abstract

Myocardial Infarction (MI) and lung cancers are major contributors to mortality worldwide. While seemingly diverse, the two share common risk factors, such as smoking and hypertension. There is a pressing need to identify bidirectional molecular signatures that link MI and lung cancer, in order to improve clinical outcomes for patients. In this study, we identified common differentially expressed genes between MI and lung cancer. Specifically, we identified 1,496 upregulated and 1,482 downregulated genes in the MI datasets. By focusing on the 1,000 most upregulated and downregulated genes in Lung Adenocarcinoma (LUAD) and Lung Squamous Cell Carcinoma (LUSC), we identified 35 genes that are common across MI, LUAD, and LUSC. Functional enrichment analysis revealed shared biological processes, such as “inflammatory response” and “cell differentiation.” The Cox proportional hazards model demonstrated a significant association between the shared genes and overall survival in lung cancer patients, as well as with smoking history in these patients. In addition, a machine learning model based on the expression of the shared genes distinguished MI patients from controls, achieving an AUROC of 0.72 and an AUPRC of 0.86. Finally, based on drug repurposing analysis, we proposed FDA-approved drugs potentially targeting the upregulated genes as novel therapeutic options for the co-occurring conditions of MI and lung cancer. Overall, our findings highlight the similarities in molecular makeup between lung cancer and MI.

## 1 Introduction

The most severe clinical manifestation of coronary artery disease (CAD) and one of the most dangerous coronary events associated with sickle cell disease (SCD) is myocardial infarction (MI) (1, 2). The two types of this pathophysiology are non-ST-elevation MI (NSTE-MI) and ST-elevation MI (STE-MI) (3). Every year, over 3 million people are diagnosed with STE-MI, and over 4 million with STE-MI, mostly in developed countries but also in developing countries (4–6). Lung cancer is the second most frequently diagnosed malignancy worldwide (7), with over 2.2 million new lung cancer cases and 1.7 million lung cancer-associated fatalities occurring annually worldwide (8). Lung carcinoma is classified into two primary types: small-cell lung cancer (SCLC) and non-small-cell lung cancer (NSCLC). The latter is further separated into three primary histological subtypes: large cell carcinoma (LCC), squamous cell carcinoma (SCC), and adenocarcinoma (ADC) (9).

Globally, acute myocardial infarction (AMI) and cancers are substantial contributors to morbidity and mortality (10). Research has shown that patients diagnosed with cancer are at a higher short-term risk of experiencing cardiovascular (CV) events, while those with acute myocardial infarction have an increased incidence of cancer (11, 12). Furthermore, cardiac complications, including myocardial infarction, heart failure, and arrhythmias, are more prevalent among cancer survivors than in the general population (13). For example, the development of adverse cardiac events in cancer patients may be influenced by inflammation, oxidative stress, and endothelial dysfunction, which are prevalent processes in both cancer and cardiovascular diseases (14). Patients with lung cancer frequently have pre-existing CVD comorbidities as a result of these overlapping risk factors. In patients with lung and bronchus cancer, hypertension, arrhythmia, CAD, dyslipidemia, and heart failure (HF) were identified as the most prevalent CV conditions (15). Moreover, the overall survival rate has been documented as lowest among patients with NSCLC and comorbid coronary heart disease, MI, or cardiac arrhythmias (16). Lung cancer has been identified as an independent risk factor for the development of CVDs, specifically CAD and MI. Consistent with the aforementioned links between the two pathologies, many risk factors are shared between cardiovascular disease (CVD) and lung cancer, including smoking, hypertension, diabetes mellitus (DM), advanced age, obesity, and racial and socioeconomic status (SES) (17, 18). These associations suggest a latent connection between AMI and lung cancer and motivate the need to identify shared mechanisms and biomarkers between the two seemingly diverse pathologies. Therefore, this study aims to identify molecular signatures underlying the bidirectional association between MI and lung cancer. Understanding the shared pathways can offer insights into overlapping disease mechanisms, improving early diagnosis and targeted therapies. Moreover, integrating these signatures may enhance personalized medicine approaches for individuals at risk of both diseases.

## 2 Methodology

### 2.1 Dataset curation and processing

We downloaded two transcriptomic datasets, GSE59867 (19) and GSE62646 (20), of MI patients from the GEO database. GSE59867 included 111 MI patients with ST-segment elevation myocardial infarction (SEMI) and 46 patients with stable coronary artery disease (CAD) as the control group. GSE62646 included 28 patients with SEMI and 14 stable CAD patients as the control. For lung cancer, we downloaded RNASeq data for both lung cancer types, LUAD (21) and LUSC (22), profiled in The Cancer Genome Atlas (TCGA). HTSeq-count and the ID/gene mapping files were downloaded from the “DATA SETS” page of the UCSC Xena Browser (23) database for both cancer types. In the case of LUAD, we obtained 527 patients and 59 normal samples, whereas, in the case of LUSC, we obtained 501 patients and 49 normal samples. For genes with multiple transcripts, we took the average read count across transcripts.

### 2.2 Characterization of DEGs

We identified the DEGs using the “limma” package (24) with default parameters with FDR <0.05. Due to the unavailability of the raw data for the MI patient-related datasets, we were unable to perform batch correction and instead relied on the identification of DEGs in each dataset independently. Normalization was done using the limma package during the process of DEG identification. Among the significant DEGs, we further selected the top 1,000 up and downregulated DEGs from each dataset (~5 percentile) based on their logFC, as the number of DEGs was vastly greater in lung cancer relative to MI and also varied substantially across the MI datasets. As there were two MI datasets, we took the union of the DEGs to represent the MI DEGs. We used R packages “ggplot2” (25) and “ggrepel” (26) to visualize the differential gene expression results as volcano plots and create Venn diagrams.

### 2.3 Gene ontology analysis

We used the clusterProfiler 4.0 (27) to characterize enriched biological processes and molecular functions. We used our list of genes (common DEGs obtained in MI, LUAD, and LUSC) as the foreground and the default human geneset as the background. The minimum and maximum gene size was set as 10 and 500, respectively, to ensure specific terms. We used “Wang” as the measuring method, and the FDR value was <=0.05. We also identified the enriched KEGG pathways using the clusterProfiler. We used the “ggplot2” R package to display up to the top 20 enriched pathways.

### 2.4 Cell survival dependency analysis

We downloaded the DepMap data (28) provided at the BROAD Institute website. The data systematically maps the genetic and chemical vulnerabilities across hundreds of cancer cell lines. The data are generated using CRISPR-Cas9 gene knockout experiment to characterize essential genes for cell survival. We downloaded the data specifically for the lung cancer cell lines with a probability score between 0 and 1, where a value >0.5 represents gene essentiality.

### 2.5 Protein–protein interaction analysis

We performed protein–protein interaction analysis and characterized interacting partners using the STRING database (29), accessible at https://string-db.org/. We provided the gene list as an input and selected “*Homo sapiens*” as the organism to search for interacting partners. We ran the tool with default parameters, selecting “full STRING network” as the network type, where the edges indicate both functional and physical protein associations, and “high confidence (0.70)” as the minimum interaction required score. We downloaded the output network images in the high-resolution “png” format.

### 2.6 Survival analysis

We used the Cox regression approach for survival association using the R package “survival” and “survminer” (30). The model was developed using gene expression, and sex and age were used as covariates. We computed the hazard ratio using a 95% confidence interval. We considered the genes and gene signatures to be significantly associated with survival if the adjusted *p*-value was <=0.05. We conducted survival analysis using common genes (up and downregulated) for both LUAD and LUSC. We also performed Kaplan–Meier (KM) curve analysis using the “Median” as the group cutoff, classifying 50% of the samples as “High” and the remaining 50% as “Low”.

### 2.7 Common gene validation using machine learning in MI patients and assessing the relationship with smoking history in lung cancer patients

We developed five different machine learning models, including support vector machine (SVM), random forest (RF), Adaboost, Gradient boost, and ExtraTree classifier, to classify MI patients from the normal population using gene expression as a feature. We performed machine learning analysis using the Python-based “Scikit” package (31). We used the stratified five-fold cross-validation for training and testing and validated the model’s performance on an independent dataset. We developed models using common 35 genes (12 up and 23 downregulated) expression as features.

To obtain the best performance, we optimize the parameters of respective classifiers during training. We used “GridSearchCV” function to get the best parameter. In the case of SVM, we tuned the parameters “kernel”, “gamma factor (g)”, and “cost factor (C)”, whereas the other four algorithms are tree-based and therefore the parameter “n_estimator” was tuned. These are the default parameters of the algorithms, and multiple values were used to obtain the best value. During each parameter tuning, stratified five-fold cross-validation was done to get the best performance. We computed the performance regarding the Area Under Receiver Operating Characteristics (AUROC) and Area Under Precision Recall Curve (AUPRC). The plots were created using the R package “pROC” (32) and PRROC (33). As a negative control, we created 10 sets of randomly selected genes and performed an analogous analysis. In the case of random gene sets, average values of the performance measures were computed.

We also assess the relationship of common genes with smoking in lung cancer patients, as smoking is one of the common risk factors among MI and lung cancer patients. We downloaded the transcriptome and clinical data for the two datasets, GSE10072 (34) and GSE50081 (35), from the GEO and grouped them into smoker (current and ex-smoker) and non-smoker categories. Next, we compared the gene expression of the common genes between the two groups and computed the Wilcoxon *t*-test to compute the significance.

### 2.8 Drug repurposing studies

We performed a drug repurposing study to propose novel drugs against our identified targets. First, we downloaded the 3D structure of the targets from the RCSB-Protein Data Bank (PDB) database (36). If they were not in the RCSB-PDB, we downloaded AlphaFold-predicted structures from the UniProt (37). Next, using the “Open Babel” software (38), we prepared the target for docking. We uploaded the prepared 3D structure of the target to the webserver “DrugRep” (39) for virtual screening and docking analysis. We selected the “protein box size” and the “centers of X, Y, and Z coordinates” after uploading the structure. Finally, we chose the library of “FDA-approved drugs” for virtual screening and downloaded the results as a zip file containing docked protein-ligand structures and the free energy.

## 3 Results

### 3.1 MI and lung cancer share common differentially expressed genes

**Figure 1:**
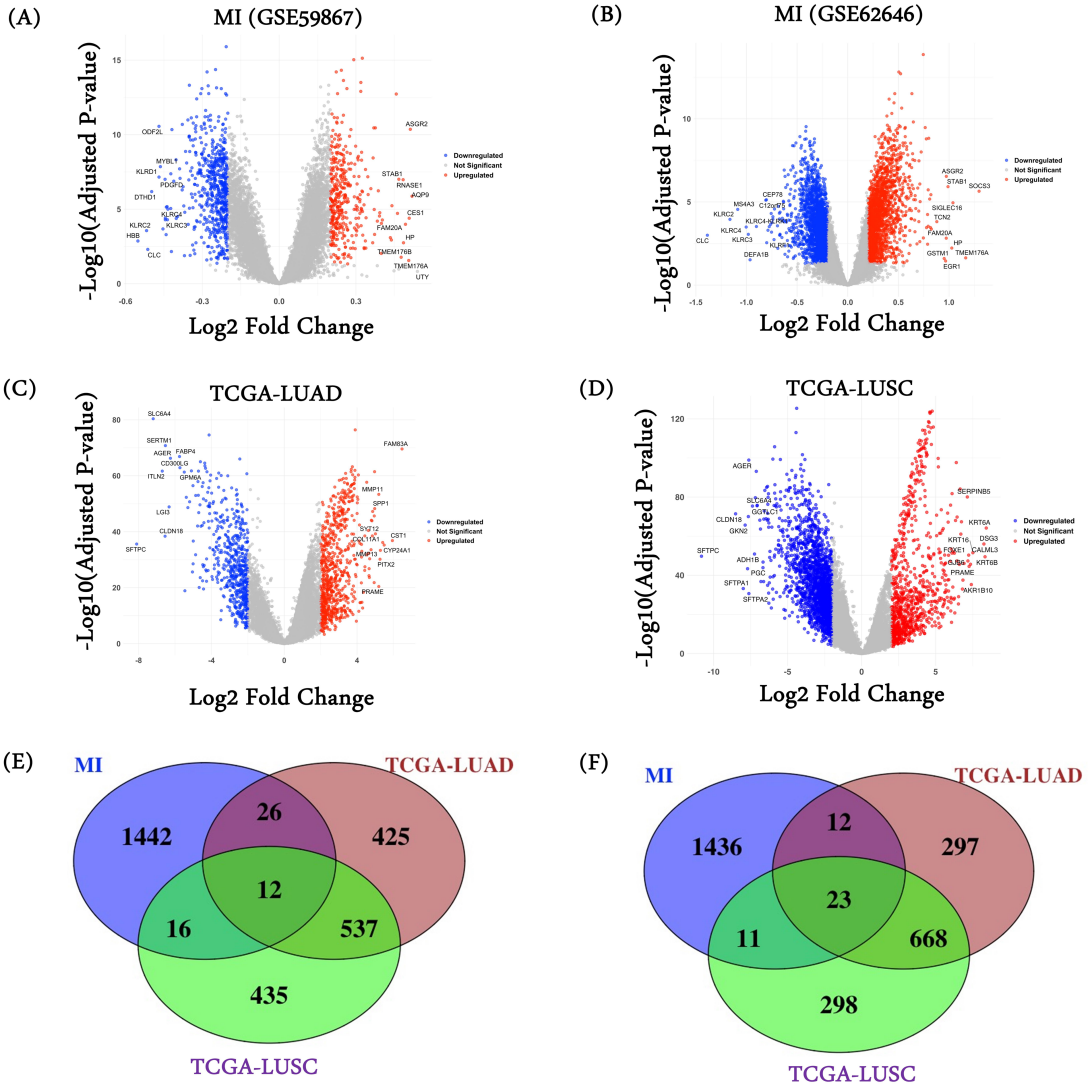
DEGs and common gene characterization. DESeq2 was used to characterize differentially expressed genes on MI datasets **(A)** GSE59867 and **(B)** GSE62646; **(C)** TCGA-LUAD and **(D)** TCGA-LUSC. Venn diagram among **(E)** MI datasets (combined), TCGA-LUAD, and TCGA-LUSC characterized upregulated genes; and **(F)** MI datasets (combined), TCGA-LUAD, and TCGA-LUSC characterized downregulated genes.

We performed differential gene expression analysis in the two MI datasets and the two lung cancer cohorts from TCGA relative to their respective controls, using “limma”. As shown in the volcano plots in Figures 1A–D, the MI datasets exhibit a narrower range of differential expression than the lung cancer datasets (consistent with broad and heterogeneous transcriptional changes in cancer), resulting in a large number of DEGs in lung cancers. We selected the top 1,000 upregulated and the top 1,000 downregulated genes in each dataset from the significant genes (FDR < =0.05). As there were two MI datasets, we merged them and retained the unique genes. In total, we obtained 1,496 genes in MI, 1000 genes in LUAD and LUSC as upregulated, and 1,482 genes in MI, 1000 genes in LUAD and LUSC as downregulated. Complete results of the differential expression analysis for all four datasets (2 MI, 1 TCGA-LUAD, and 1 TCGA-LUSC) are provided in Supplementary Tables S1–S4. Next, we overlapped the three sets of differentially expressed genes to obtain the common up and downregulated genes (Figures 1E,F). In total, we obtained 35 common genes (12 upregulated genes and 23 downregulated genes) among MI, LUAD, and LUSC. Of note, the expected overlaps based on random expectation are 3.74 and 3.70. A list of these common genes is provided in Supplementary Table S5.

### 3.2 Functional enrichment analysis elucidates common pathways between MI and lung cancer

**Figure 2:**
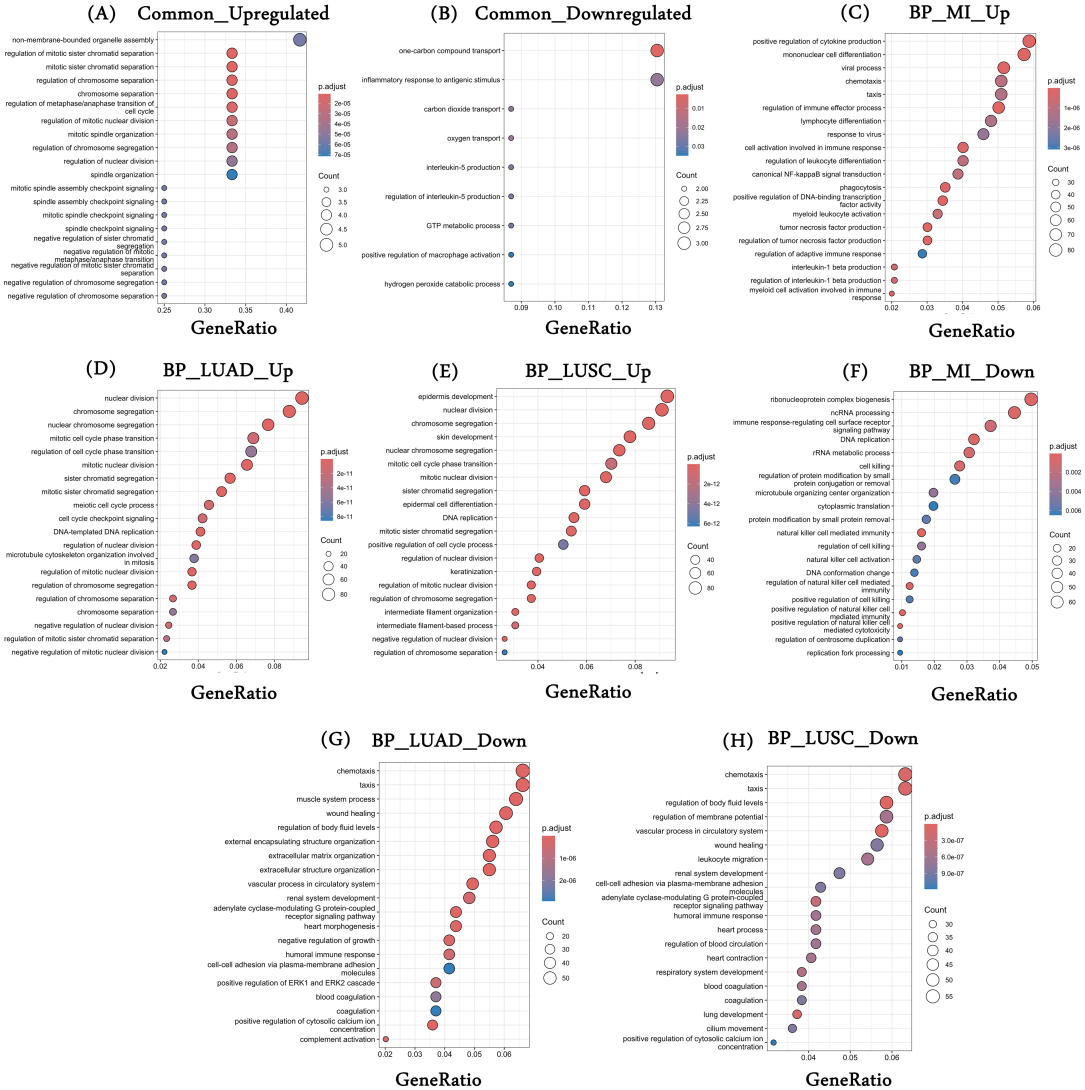
Gene enrichment analysis. **(A,B)** Represent the top 20 statistically significant enriched biological processes associated with common upregulated and downregulated genes, respectively; **(C–E)** Represent the top 20 statistically significant enriched biological processes associated with **(C)** upregulated genes in MI datasets (combined); **(D)** upregulated genes in TCGA-LUAD; and **(E)** upregulated genes in TCGA-LUSC and **(F–H)** represents the top 20 statistically significant enriched biological processes associated with **(F)** downregulated genes in MI datasets (combined); **(G)** downregulated genes in TCGA-LUAD; and **(H)** downregulated genes in TCGA-LUSC. *BP, biological processes; MI, myocardial infarction; LUAD, lung adenocarcinoma; LUSC, lung squamous cell carcinoma.

We performed the functional enrichment analysis using the DEGs characterized in each diseased condition and the DEGs common to all three conditions. First, the 12 common upregulated genes were majorly enriched for the cell cycle-associated processes, such as “chromosome separation,” “spindle organization,” “spindle checkpoint signaling,” etc. (Figure 2A). Independent studies have demonstrated the role of cell cycle-associated processes in MI and lung cancer patients. Although there are no direct associations of the cell cycle with the initiation of MI, our observations may reflect post-MI healing, stress response, immune response, etc. For example, Fu et al. showed that post-MI, fibroblasts show higher cell differentiation and proliferation for long-term tissue remodeling and wound healing (40). Similarly, Curaj et al. showed the role of immune-mediated cell cycle regulation where neutrophils modulate fibroblast function and promote healing post-MI (41). Therefore, there is a growing interest in understanding the cell cycle regulation in MI patients. In the case of cancers, the role of cell cycle regulation is well established, and it has been reported that the loss of the ability of cells to proliferate in a controlled fashion leads to cancer (42, 43). Supplementary Table S6 provides a comprehensive list of enriched biological processes among the 12 common upregulated genes.

Similarly, the common 23 downregulated genes were enriched for “inflammatory response to antigenic stimulus,” “oxygen transport,” “interleukin-5-production,” etc. (Figure 2B). Oxygen transport plays an important role in the development and progression of MI and many cancers, including lung. For example, it is intricately linked to erythrocyte differentiation, which has been observed in both conditions previously. In MI patients, the heart undergoes severe damage and stress, and the production of inflammatory cytokines such as TNF-*α*, IL-1β, and IL-6 takes place. These cytokines inhibit the process of erythropoiesis by remodeling the bone marrow microenvironment (44). In the case of lung cancer, a hypoxic environment alters the normal hematopoiesis as cancer cells compete for oxygen, leading to a less favorable environment for erythrocyte formation and differentiation (45). Similarly, the role of IL-5 in MI and lung cancer has been established (46, 47). A complete list of enriched biological processes associated with common downregulated genes is provided in Supplementary Table S7.

We also looked at the enriched processes associated with individual disease conditions—MI and the two lung cancers. Processes such as “positive regulation of DNA-binding transcription factor activity,” “positive regulation of cytokine production,” and “positive regulation of NIK/NF-kappaB signaling” are primarily enriched for the upregulated DEGs in MI (Figure 2C). DEGs that were upregulated in LUAD have been linked to cell cycle-associated processes, such as “nuclear division,” “chromosome segregation,” “cell cycle checkpoint signaling,” and more (Figure 2D). Similarly, in LUSC, processes such as “epidermis development,” “mitotic cell cycle phase,” etc., were associated with the upregulated genes (Figure 2E). In the case of downregulated genes, MI was broadly enriched for the immune-related processes, such as “immune response-regulating cell surface receptor signaling pathway,” “natural killer cell mediated immunity,” etc. (Figure 2F). For LUAD, downregulated genes were enriched for the processes such as “wound healing,” “extracellular matrix organization,” “heart morphogenesis,” etc. (Figure 2G), and finally, DEGs downregulated in LUSC were enriched for the processes such as “vascular processes in circulatory system,” “heart contraction,” “lung development” and others (Figure 2H). The top 20 processes are shown in Figures 2A–H, and the list of all enriched biological processes is provided in Supplementary Tables S8–S10.

The KEGG pathway analysis was also performed to characterize the enriched pathways associated with the disease-specific and common DEGs across diseased conditions. We observed only two significant pathways associated with the commonly upregulated genes, i.e., “Cell cycle” and “Oocyte meiosis.” In the case of MI, upregulated DEGs were majorly enriched for “Lysosome,” “Osteoclast differentiation,” “NOD-like receptor signaling pathway,” “TNF signaling pathway,” etc. Similarly, upregulated genes in LUAD and LUSC show enrichment of common pathways such as “cell cycle,” “Oocyte meiosis,” and the “Fanconi anemia pathway,” as well as unique pathways such as “Motor proteins” in LUAD and “p53 signaling pathway,” “Retinol metabolism,” etc. in LUSC. Supplementary Tables S11–S14 include the complete list of enriched KEGG pathways for the common and disease-specific upregulated genes. We also performed a similar analysis with the downregulated genes, and the complete results for enriched biological processes are provided in Supplementary Tables S15–S17. Similarly, enriched KEGG pathways associated with common and disease-specific downregulated genes are provided in Supplementary Tables S18–S21.

### 3.3 Shared genes are associated with cell survival

CRISPR-Cas9 knockout-based DepMap data were downloaded for the lung cancer-specific cell lines. Median dependency scores across cell lines for the shared genes were computed. In the case of 12 upregulated genes, *CDCA8* and *E2F2* were associated with cell survivability with probability scores of 0.99 and 0.95, respectively. These genes play a key role in cell division, as *CDCA8* is associated with genome transfer during cell division, whereas *E2F2*, a transcription factor, controls cell cycle processes. The role of these genes is well-established in lung cancer (48, 49). Similarly, in the case of 23 downregulated genes, *CPA3, GIMAP7*, and *SLC14A1* show the cell survival dependencies with a probability of 0.99, 0.94, and 0.93, respectively. The *CPA3* gene is expressed in mast cells and is associated with maintaining homeostasis. The role of this gene in lung cancer has been shown in previous studies (50). Similarly, *GIMAP7*, a member of the GTPase family, is associated with regulating immune cell infiltration and the development and progression of multiple cancers, including lung cancer. However, given that DepMap is based on cell lines, the cell-endogenous role of *GIMAP7* is unclear. Li et al. show that the downregulation of *GIMAP7* is associated with poor prognosis and aggressive behavior of lung adenocarcinoma (51). Similarly, Zhou et al. show the association of down expression of *SLC14A1* with poor prognosis and progression of NSCLC (52). The association of downregulation of GIMAP7 and *SLC14A1* with worse prognosis suggests a role independent of cell viability. The DepMap probability score for the DEGs is provided in Supplementary Table S22.

### 3.4 Protein–protein interaction analysis elucidates diverse interacting partners of key targets

**Figure 3:**
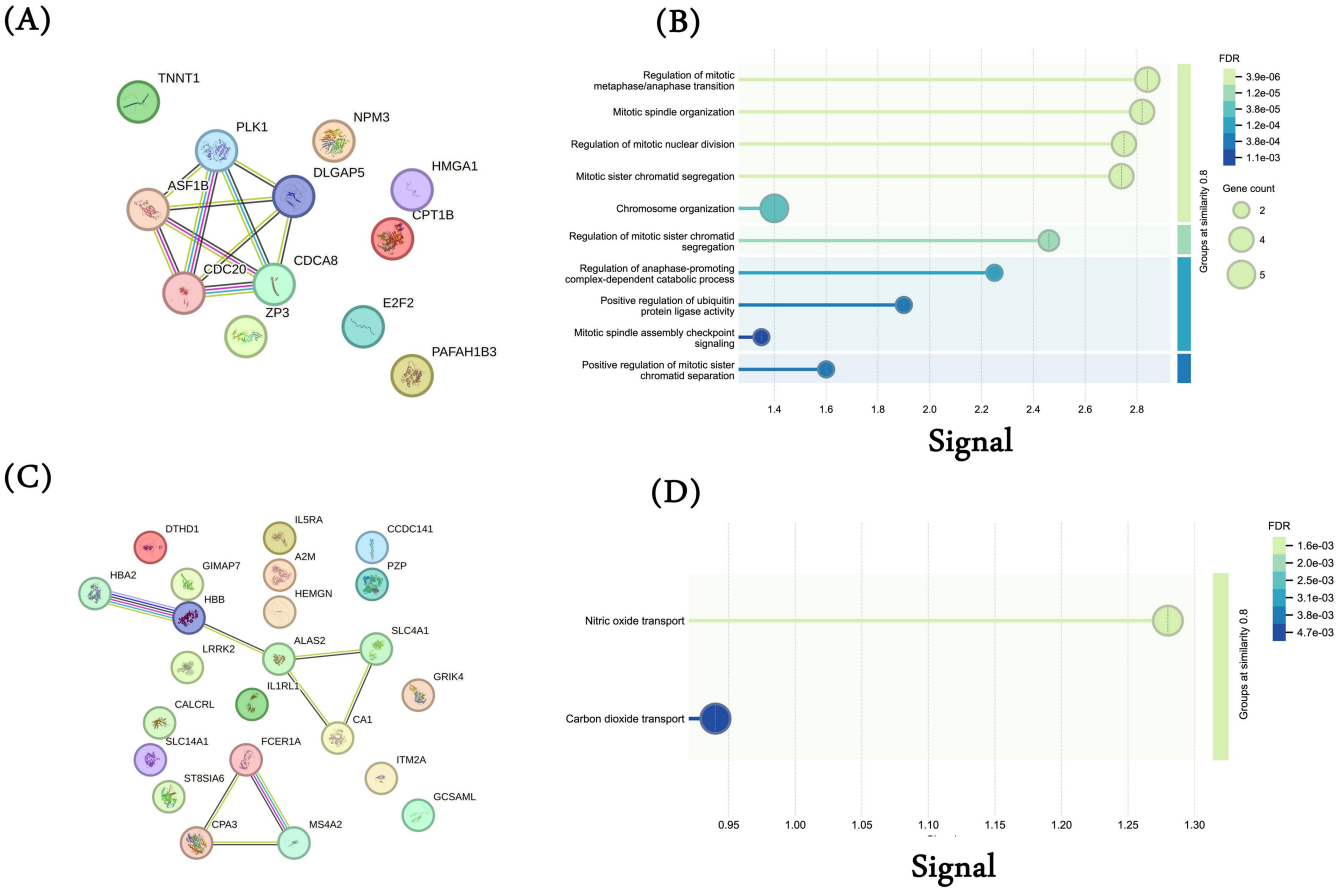
Protein–protein interaction (PPI) analysis. PPI network generated using STRING database for the 12 common upregulated genes among MI, TCGA-LUAD, and TCGA-LUSC datasets. Each gene was submitted individually in the STRING database and the interaction network was downloaded. **(A)** Common 12 upregulated genes; **(B)** biological processes for common 12 upregulated genes (gene ontology); **(C)** common 23 downregulated genes; **(D)** biological processes for common 23 downregulated genes (gene ontology).

Here, we aim to discover protein modules among the common up and downregulated genes and their additional interaction partner to gain further insights into additional proteins and processes. We conducted a protein–protein interaction (PPI) analysis of common DEGs using the STRING database. First, we performed the analysis using the 12 common upregulated DEGs and observed an interaction among the genes *PLK1, ASF1B, CDC20, CDCA8,* and *DLGAP5* (Figure 3A). These genes are associated with cell cycle-associated processes, as shown in Figure 3B. We also conducted a similar analysis using 23 common downregulated genes and observed two interaction modules, one among genes *HBA2, HBB, ALAS2, SLC4A1,* and *CA1* and the other in between *FCER1A, CPA3,* and *MS4A2* (Figure 3C). Enrichment analysis for the first interaction networks was enriched for “Nitric oxide transport” and “Carbon dioxide transport” (Figure 3D).

We then analyzed additional interaction partners of these common upregulated (Supplementary Figure S1) and downregulated genes (Supplementary Figure S2). While there are some interactions among the up and downregulated genes, this analysis also suggests that these genes may affect multiple independent processes and may correspond to heterogeneous phenotypes.

### 3.5 Key genes are associated with overall survival in lung cancer patients

Here, we assessed whether the common genes are associated with patient prognosis, specifically in lung cancer, where the requisite data are available in TCGA. Using the Cox regression approach for overall survival analysis for the LUAD and LUSC cohorts in TCGA, we observed 4 of 12 genes, namely *CDC20, DLGAP5, HMGA1,* and *PLK1,* were associated with poor survival of LUAD cancer patients (Table 1). However, none of the genes show a significant survival association for LUSC cancer patients. Controlling for age and sex as covariates did not change these results. Similarly, in the case of downregulated genes, significant survival association was seen for *SLC14A1*, *MS4A2,* and *HEMGN*.

**Table 1:** The Cox regression results for 12 common upregulated genes.

Genes	HR	p.value	CI.lower	CI.upper	p.adjusted
LUAD cohort					
ASF1B	1.160058843	0.0128348	1.03202767	1.30397329	0.15401757
CDC20	1.167130301	0.00176356	1.05938848	1.28582966	0.02116276
CDCA8	1.159419166	0.00899113	1.03763265	1.29549972	0.10789357
CPT1B	0.952927027	0.3174116	0.8669793	1.04739516	1
DLGAP5	1.225788067	6.13E-05	1.1096253	1.35411151	0.00073562
E2F2	1.06686299	0.25535455	0.95427513	1.19273426	1
HMGA1	1.217319908	0.00081233	1.08496571	1.3658199	0.00974793
NPM3	1.063617043	0.37819143	0.92728183	1.21999717	1
PAFAH1B3	1.128208335	0.05669227	0.99656942	1.27723571	0.68030728
PLK1	1.253633377	2.73E-05	1.12798533	1.39327756	0.0003275
TNNT1	1.064795367	0.01635512	1.01159689	1.12079148	0.19626144
ZP3	1.083524015	0.15398963	0.97037733	1.20986369	1
LUSC cohort					
ASF1B	0.981040967	0.77867073	0.8584519	1.12113606	1
CDC20	1.057784384	0.43558328	0.91847411	1.21822466	1
CDCA8	1.030475775	0.68029215	0.89335529	1.18864279	1
CPT1B	1.036125181	0.49023218	0.93676258	1.14602719	1
DLGAP5	0.95405697	5.01E-01	0.83193545	1.09410496	1
E2F2	1.01179911	0.86278041	0.88577796	1.15574951	1
HMGA1	1.044064356	0.56927388	0.89998133	1.21121444	1
NPM3	0.958106971	0.57041706	0.82645041	1.1107369	1
PAFAH1B3	1.00736051	0.90464814	0.89345985	1.13578153	1
PLK1	1.066686745	4.06E-01	0.91606754	1.24207066	1
TNNT1	1.008069882	0.78760635	0.95081739	1.06876978	1
ZP3	0.954804523	0.44044292	0.84895446	1.07385227	1

The Cox regression model was developed using gene expression with age and gender as covariates. The analysis was performed for both LUAD and LUSC cohorts in TCGA. HR, hazard ratio; CI, confidence interval.

The key roles that these genes play in tumorigenesis further explain this observation. Miao et al. have shown the role of *CDC20* in the migration, invasion, and proliferation of lung adenocarcinoma cells *in vitro* (53). Similarly, Chen et al. reported that *DLGAP5* upregulates *PLK1,* promotes cell proliferation in LUAD patients, and exhibits a strong correlation with unfavorable prognosis (54). Similarly, Zhou et al. show that *SLC14A1* downregulation is associated with poor survival outcomes in non-small cell lung cancer (NSCLC). In another study, Zheng et al. have shown that high expression of *MS4A2* genes is beneficial for the overall survival of LUAD patients (55). *MS4A2* is associated with immune-infiltrating cells such as B cells, CD4^+^ T cells, CD8^+^ T cells, macrophages, etc., which play an important role in the immune response in patients. Interestingly, in the case of LUSC, we did not observe any gene associated with survival. This may be because LUAD and LUSC are different in their genetic landscape, tumor microenvironment (TME), and therapeutic response (56, 57). For example, LUAD is primarily driven by oncogenic mutations in genes, such as *EGFR, KRAS, AL, BRAF,* and *MET,* which are associated with the activation of MAPK, PI3K/AKT, and JAK/STAT signaling pathways, whereas LUSC is driven by mutations in *TP53, NFE2L2, NOTCH1,* and *SOX2,* affecting cell cycle regulators (58). Unlike LUAD, LUSC relies more on squamous differentiation pathways. Furthermore, LUAD shows higher CD8+ T and dendritic cell infiltration compared to LUSC, where higher infiltration of neutrophils and macrophages is observed (59, 60). These differences in the two subtypes may partly explain the subtype-specific overall survival. For example, in LUAD, *CDC20,* which is an anaphase-promoting complex (APC/C), is strongly linked to EGFR and KRAS-mutant LUAD and is associated with poor prognosis (61), whereas LUSC relies on other regulators such as *CCND1* and *RB1* (62).

**Figure 4:**
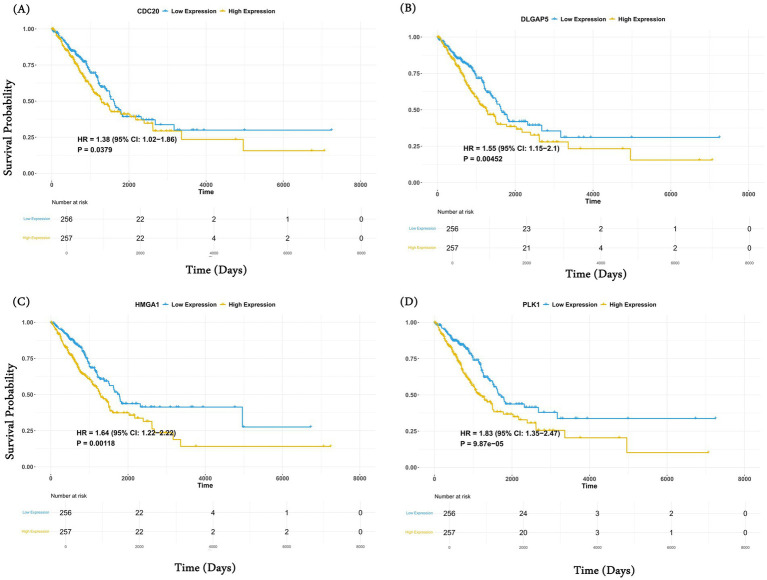
Common genes are associated with overall survival analysis. Kaplan–Meier (KM) curve of the four significant upregulated genes **(A)** CDC20; **(B)** DLGAP5; **(C)** HMGA1; and **(D)** PLK obtained after the Cox regression analysis. For each gene, patients were stratified into high (50%) and low (50%) categories based on median expression.

The complete results for the Cox regression analysis for 12 common upregulated genes in LUAD and LUSC cohorts are provided in Table 1. The KM curve for the above-identified significant upregulated genes is shown in Figures 4A–D. Similarly, for downregulated genes, the results for Cox regression analysis are provided in Supplementary Tables S23–S24 for LUAD and LUSC, respectively, and the KM curve for the significant downregulated genes as per the Cox regression results is shown in Supplementary Figure S3.

### 3.6 Common genes can classify MI patients from normal with high accuracy

**Figure 5:**
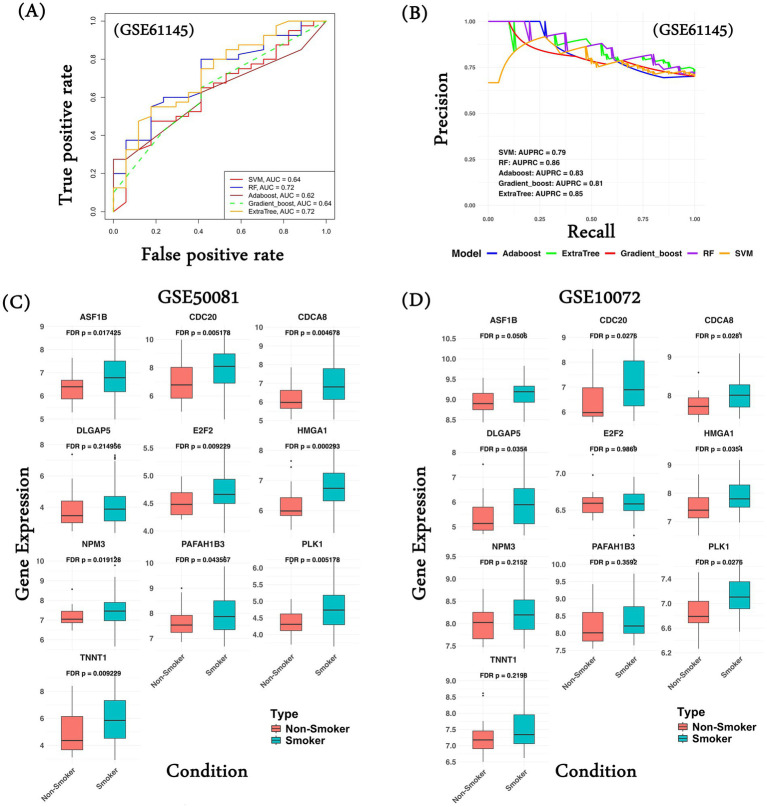
Common gene signature performance on validation datasets. **(A)** Performance of various ML models using common 30 gene expression in terms of AUROC values; **(B)** Performance of various ML models using common 30 gene expression in terms of AUPRC values; **(C)** Gene expression comparison of common upregulated genes in the lung cancer patients classified as smoker and non-smoker in the dataset GSE50081; **(D)** Gene expression comparison of common upregulated genes in the lung cancer patients classified as smoker and non-smoker in the dataset GSE10072. ML model performance was computed in the form of ROC curves, gene expression comparison was made in the form of a boxplot, and the Wilcoxon test was performed as a measure of statistical significance.

Here, we assessed the extent to which the common differential genes’ expression can distinguish MI patients from normal controls in an independent dataset. We trained and tested five machine learning models (SVM, RF, Adaboost, Gradient Boost, and ExtrTree) to classify MI patients from normal using the gene expression of the common genes characterized on the datasets used in the study for MI (see Methods section). Next, we validated the performance of the models on an independent dataset, GSE61145 (63). Five genes (one upregulated and four downregulated) were absent in GSE61145. Among all methods, the RF model based on the 30 common genes achieved the maximum AUROC of 0.72 (Figure 5A). The performance of the five ML models along with their best parameters is provided in Supplementary Table S25. As a negative control, random sets of 30 genes achieved an AUROC ranging from 0.49 to 0.56 (Supplementary Table S26). The AUPRC for the RF model was 0.86 (expectation is 0.3; Figure 5B). To assess the impact of missing five genes on the robustness of the model performance, we performed an internal five-fold cross-validation on the dataset GSE59867 using data from both 30 and 35 genes. As shown in Supplementary Table S27, models trained using both sets of genes show similar performance. These observations suggest that our common gene set can classify MI and non-MI patients with reasonable accuracy, well above random expectations.

In an additional analysis, we tried to assess the relationship of the common gene signatures with smoking history in lung cancer patients, as smoking is one of the common risk factors among MI and lung cancer. We hypothesize that the common genes exhibit differential expression in lung cancer patients with a smoking history relative to non-smokers. We compared the gene expression of the 10 upregulated genes (*CPT1B* and *ZP3* expression were not available) and observed that all the genes (except *DLGAP5*) show significantly high gene expression among the patients with a smoking history in GSE50081 (Figure 5C). Similarly, in another dataset, GSE10072, 6 of 10 genes show significantly higher gene expression in the smoker group (Figure 5D). We performed a similar analysis of the common downregulated genes. In the case of dataset GSE50081, the majority of the genes show significantly higher gene expression in the non-smoker group compared to the smoker group (Supplementary Figure 4A); however, in the case of GSE10072, we do not observe significant gene expression in the non-smoker category (Supplementary Figure 4B). This analysis supports our hypothesis that lung cancer patients with a smoking history have higher chances of developing MI compared to those without a smoking history.

### 3.7 Drug repurposing analysis potential drugs against MI and lung cancer

Addressing the currently limited therapeutic options for MI and lung cancer, here, we conducted a drug repurposing analysis to identify drugs that can potentially target the 12 common upregulated genes associated with the disease biology in both MI and lung cancer. First, for the protein products of the 12 genes (from UniProt), we download their 3D structure from the RCSB-PDB database. For genes lacking the PDB ID or full-length 3D structure, we download the AlphaFold predicted structure from the UniProt database (see Supplementary Table S28 for details). We then used Open Babel software to process and refine the structures by removing heteroatoms and metal ions, among other things. Next, using the DrugRep server^1^, we identified the binding pockets on the protein surface and chose the one with the largest volume because it has more active site residues that can interact during docking (Supplementary Table S29). Next, we selected the “FDA-approved drugs” library for screening against our target and submitted it for docking, where the server uses the AutoDock Vina tool for docking. As a result, we obtained the top 100 drugs for each protein, each represented by a “DrugBank ID” and their free energy value (Supplementary Table S30). We mapped the top three hits with the highest free energy using the DrugBank database to obtain the drug names. Table 2 provides a list of the top three drugs for each target.

**Table 2:** List of 12 common upregulated genes and top three drugs mapped to them after drug repurposing analysis.

Sr. No.	Target	Top 3 drugs
1	*ASF1B*	Lomitapide, Nebivolol, Conivaptan
2	*CDC20*	Saquinavir, Digoxin, Rimegepant
3	*CDCA8*	Phenytoin, Tolnaftate, Cyclandelate
4	*CPT1B*	Dutasteride, Conivaptan, Rimegepant
5	*DLGAP5*	Pimozide, Ubrogepant, Drospirenone
6	*E2F2*	Conivaptan, Venetoclax, Ponatinib
7	*HMGA1*	Nilotinib, Conivaptan, Regorafenib
8	*NPM3*	Estradiol, benzoate, Ergotamine, Adapalene
9	*PAFAH1B3*	Lifitegrast, Dihydroergotamine, Lomitapide
10	*PLK1*	Tadalafil, Hexafluronium, Venetoclax
11	*TNNT1*	Capmatinib, Entrectinib, Imatinib
12	*ZP3*	Darifenacin, Hydromorphone, Cyproheptadine

## 4 Discussion

Lung cancer is one of the leading cancers, affecting 2,480,675 people (both men and women) worldwide. As per the International Agency for Research on Cancer (IARC), lung cancer was the leading cause of cancer deaths, with 1.8 million deaths (18%) in 2020. Smoking is the leading cause of lung cancer, as it is responsible for nearly 85% of all cases (15). One of the reasons for the high mortality rate of cancer is its diagnosis at an advanced stage, where treatment options are limited. If diagnosed early, the survival rate can be dramatically improved. Lung cancer has been hypothesized to be associated with increased cardiovascular diseases, especially coronary heart disease (64), stroke (65), and MI (66). MI (also known as heart attack) is a type of CVD that is prevalent worldwide and is associated with significant morbidity and mortality. MI occurs due to a decreased or complete cessation of blood flow to the myocardium. A large cohort-based study known as “INTERHEART” conducted across 52 countries identified several risk factors associated with MI, which include smoking, hypertension, abnormal lipid profile/blood lipoprotein (ApoB/ApoA1), and diabetes mellitus (67, 68).

Numerous studies have observed a direct or indirect association between lung cancer and MI (18, 66, 69, 70). They share similar risk factors, such as smoking, obesity, inflammation, and so on; however, because of their complex and distinct pathophysiologies, analyzing both conditions has been challenging. In this study, we tried to address this issue by analyzing MI and lung cancer together. We performed differential gene expression analysis using genome-wide transcriptome data from GEO for MI patients and TCGA for lung cancer (LUAD and LUSC). We characterized DEGs (12 up- and 23 downregulated) as common in all three conditions and performed several downstream analyzes. The GO analysis reveals an enrichment of pathways and processes found to be common among both diseased conditions. For example, common-up genes were found to be enriched for metabolic processes, immune-related processes, cell death regulation, and IL-5-mediated signaling pathways, indicating a possible alteration in the metabolic and immune landscape in the tumor microenvironment and the cardiovascular system.

We observed that shared genes mechanistically bridge the gap between MI and lung cancer. For example, *PLK1* (Polo-like kinase 1) gene overexpression has been associated with cell proliferation of tumor cells in cancer (71) and cardiomyocytes and reducing apoptosis in MI (72). *PLK1* inhibition induces mitotic arrest and apoptosis in cancer cells, highlighting its potential as a therapeutic target. Similarly, *E2F2* (E2F transcription factor 2) is associated with gene regulation not only in lung cancer but also in MI. It controls cell proliferation, invasion, and migration in lung cancer (49), whereas in MI, dysregulation of *E2F2* leads to cardiomyopathies (73). In addition to cell cycle genes, we also observed *HMGA1* (High Mobility Group AT-Hook 1), a non-histone chromatin binding protein associated with altering chromatin structure and gene expression. Its role in tumor progression and metastasis in lung cancer is established (74). In the case of MI, the gene plays a similar role, i.e., regulating gene transcription and chromatin remodeling and its overexpression is associated with cardiomyocyte inflammation and apoptosis (75).

Similarly, PPI analysis using STRING reveals the association of common genes with other proteins that regulate a diverse range of relevant processes. Next, we looked for the survival association of the genes to predict prognosis in LUAD and LUSC patients and observed a significant association of the genes, especially up genes in the LUAD cohort. We also show that a machine learning model, based on the common genes’ expression, can differentiate MI patients from controls with an AUROC of 0.72 and AUPRC of 0.86 compared to the negative control. This result indicates the potential use of these genes as biomarkers for early diagnosis and risk assessment. We further validated our characterized signatures on external datasets where we compared the gene expression pattern in lung cancer patients classified as smokers or non-smokers. This is an important analysis, as “smoking” is one of the common risk factors for MI and lung cancer. As hypothesized, we observed significantly higher gene expression in the smoker group than in the non-smoker group. Although the current analysis is based on gene expression, future studies need to take other correlates into account, such as age and body mass index (BMI), to obtain more refined insights. Finally, we performed a drug repurposing analysis against the 12 common upregulated genes and proposed novel drugs.

The upregulation of 12 genes that are common to both lung cancer and MI suggests a converging pathogenesis. This forms a rationale for future work in the prediction of CV risk in patients with lung cancer, taking into account race and age-specific populations, which will likely increase the predictive value of the computational platform with capabilities to predict patterns and processes associated with not only disease susceptibility but also morbidity and mortality.

Given that the current study suggests a potential link between MI and lung cancer pathophysiology, it is important to address certain limitations. First, the current study is based on a relatively small dataset with limited geographical diversity; going forward, it will be important to extend the current analysis to a larger and more diverse cohort. Second, the current study analyzes only transcriptome data and needs integration of other omics data, such as proteomics and metabolomics, for a more complete view of disease mechanisms. Third, we need to experimentally validate the proposed drugs. Finally, we need to collect data where the patient with lung cancer has a previous history of MI or vice versa. Overall, the current study directs us to how we can leverage the commonality of the two diseases to improve our understanding and management, ultimately improving patient outcomes.

## 5 Conclusion

The identification of significant upregulated genes from myocardial infarction (MI) and lung cancer, specifically LUAD and LUSC, suggest a shared mechanism of pathophysiology. Such genes could serve as potential biomarkers in early detection and risk stratification in both diseases, thereby improving triaging of patients and clinical decision-making. The application of ML models further supports the potential of these genetic signatures to classify patients with high accuracy, providing a promising tool for integrating precision medicine, which will provide a basis for precision public health as well. This paves the way to validate such biomarkers in larger and more diverse populations in a race-specific manner for further assessment of the broader applicability of biomarkers, pursuing an ultimate goal to improve early detection, personalized treatments, and improved patient outcomes for both cardiovascular and lung cancer care. Moreover, our study provides a robust underpinning to further development in predicting the bi-directional risk of MI and lung cancer patients incorporating ethnicity- and age-specific variables. Demographic tailoring of such predictions will enhance the accuracy of algorithms using computational models that not only identify susceptibility to disease but also morbidity and mortality risks.

## Data Availability

This paper analyzes existing, publicly available data. The accession numbers for the datasets used are listed in the key resources table. All the codes, datasets used to develop the machine learning models for each class is provided at out GitHub repository accessible at https://github.com/agrawalpiyush-srm/MI_LungCancer. Further information can be provided upon reasonable request to the corresponding author.

## Footnotes

^1^ https://cao.labshare.cn/drugrep/
