# Supplementary material for: Molecular signatures bidirectionally link myocardial infarction and lung cancer
Source: Front Med (Lausanne). 2025 Apr 9;12:1576375. doi: 10.3389/fmed.2025.1576375 (PMC12014433; doi:10.3389/fmed.2025.1576375)
Supplement: Supplementary file 1 [file Supplementary_file_1.docx]

**Molecular Signatures Bidirectionally Link Myocardial Infarction and Lung Cancer**

Dhruva Nandi^1^, Rajiv Janardhanan^1^, Sridhar Hannenhalli^2*^, Piyush Agrawal^1*^

1. Division of Medical Research, SRM Medical College Hospital & Research Centre, SRMIST, Kattankulathur, Chennai, Tamil Nadu, India
2. Cancer Data Science Laboratory, NCI, NIH, Bethesda, USA

*** Corresponding Author**

Piyush Agrawal, Ph.D.

Division of Medical Research, SRM Medical College Hospital & Research Centre, SRMIST, Kattankulathur, Chennai, India-603203

**Email:** [piyusha@srmist.edu.in](mailto:piyusha@srmist.edu.in); [apiyush74@gmail.com](mailto:apiyush74@gmail.com)

Sridhar Hannenhalli

Cancer Data Science Laboratory, NCI, NIH, Bethesda, USA

**Email:** [sridhar.hannenhalli@nih.gov](mailto:sridhar.hannenhalli@nih.gov)


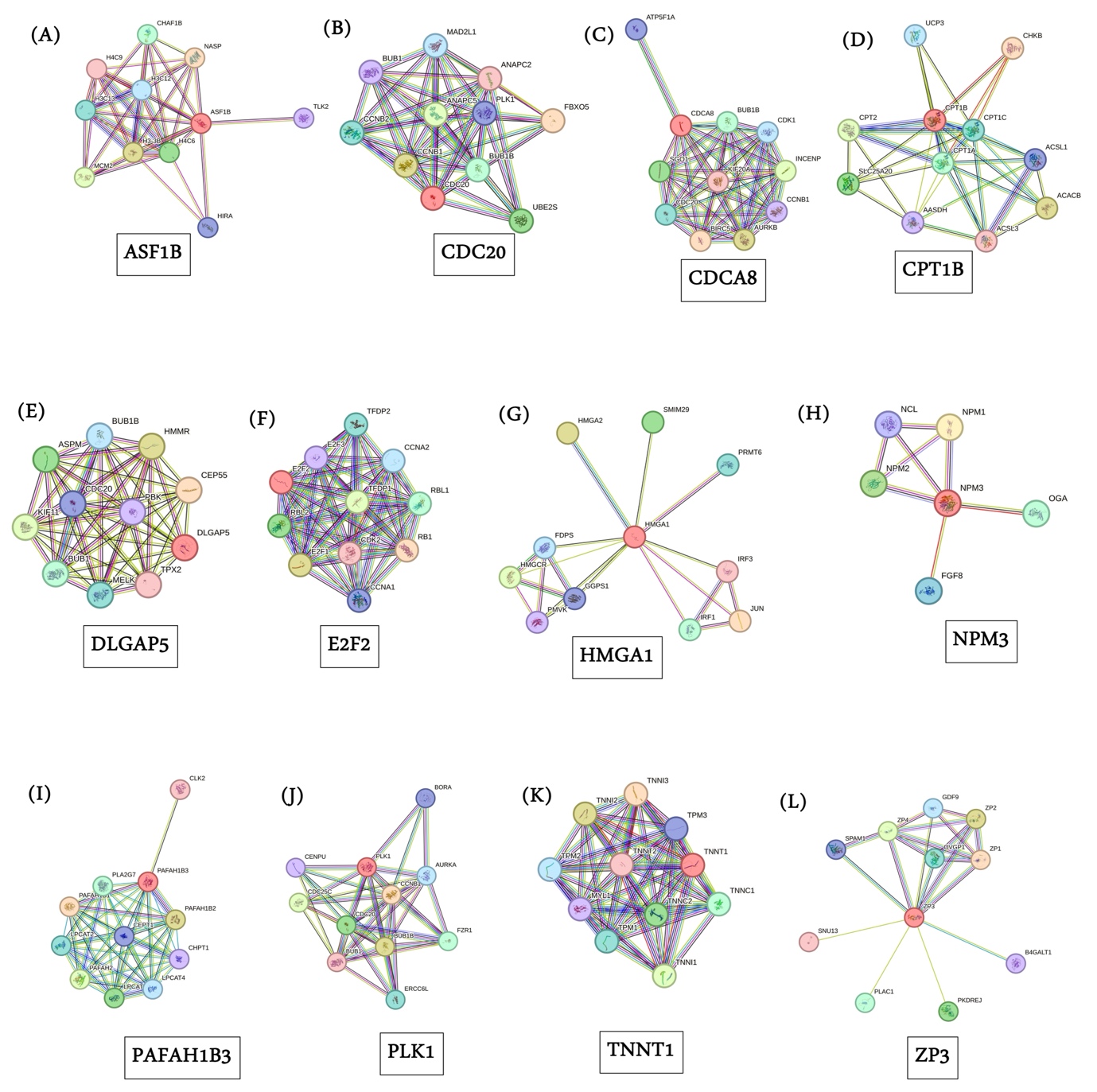


**Supplementary Figure S1.** **Protein-Protein Interaction (PPI) Analysis.** PPI network generated using STRING database for the common 12 upregulated genes among MI, TCGA-LUAD and TCGA-LUSC datasets. Each gene was submitted individually in the STRING database and the interaction network was downloaded.


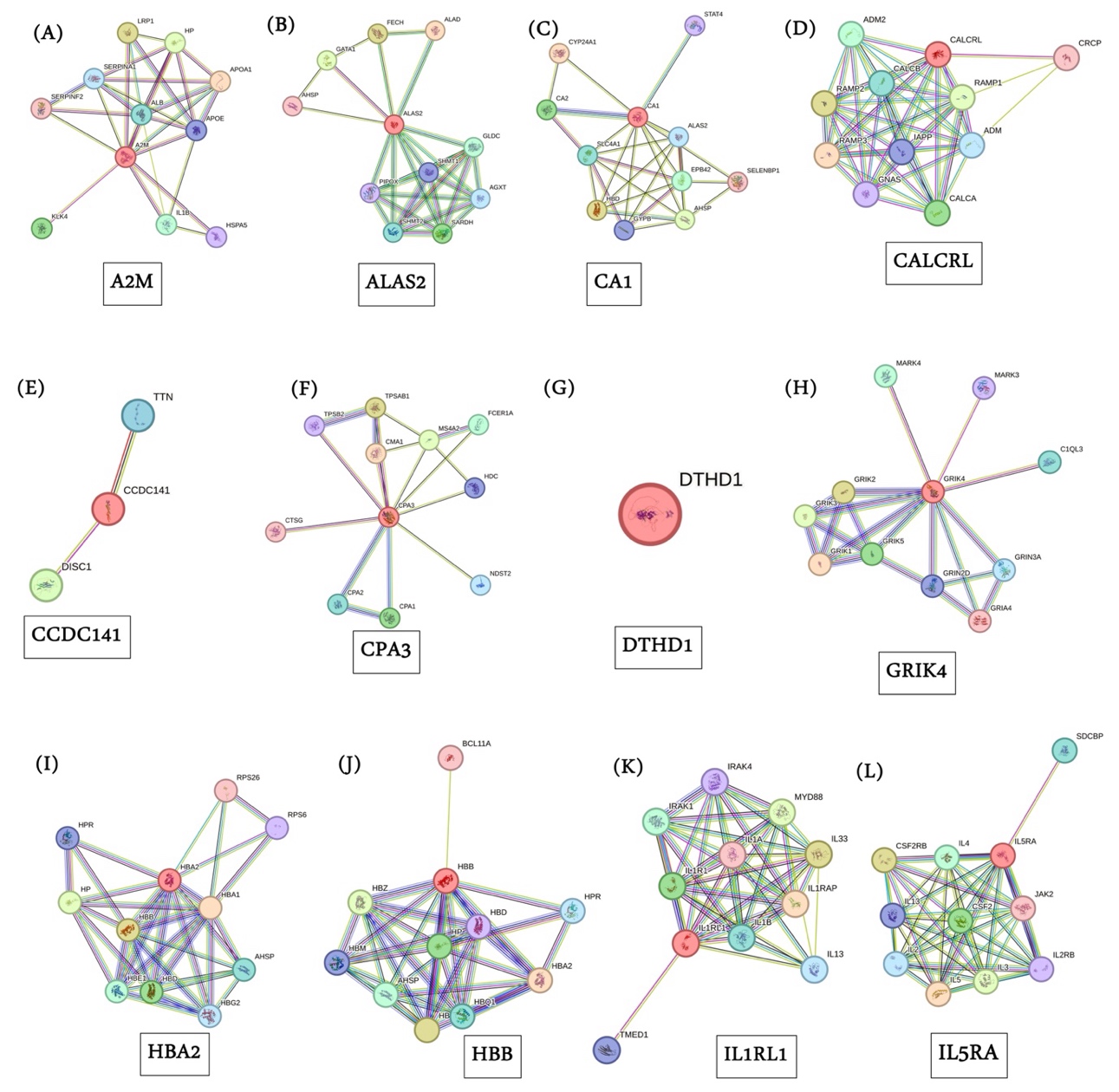


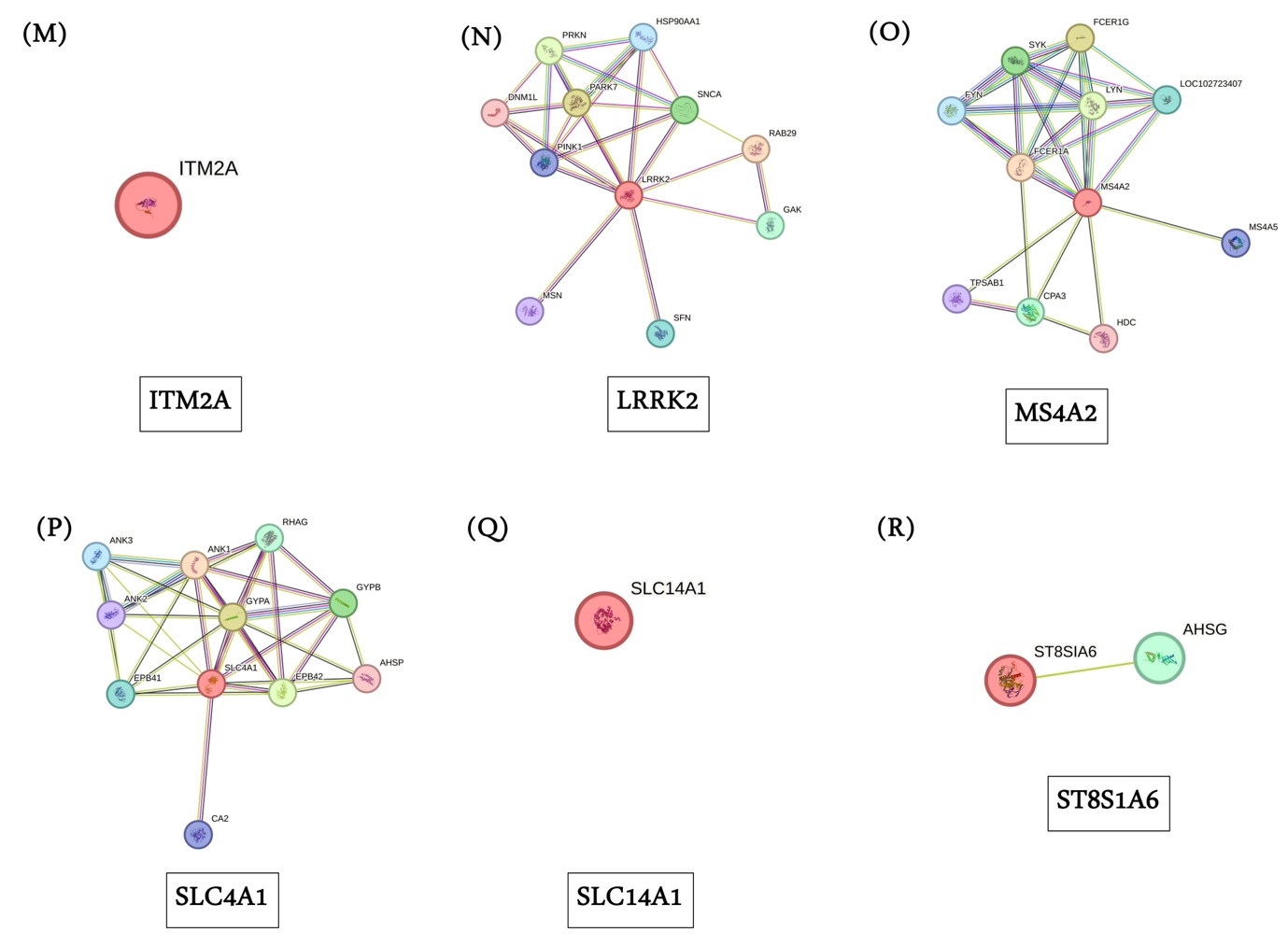


**Supplementary Figure S2.** PPI network generated using STRING database for the common 23 downregulated genes among MI, TCGA-LUAD and TCGA-LUSC datasets. Each gene was submitted in the STRING database individually and the interaction network was downloaded.


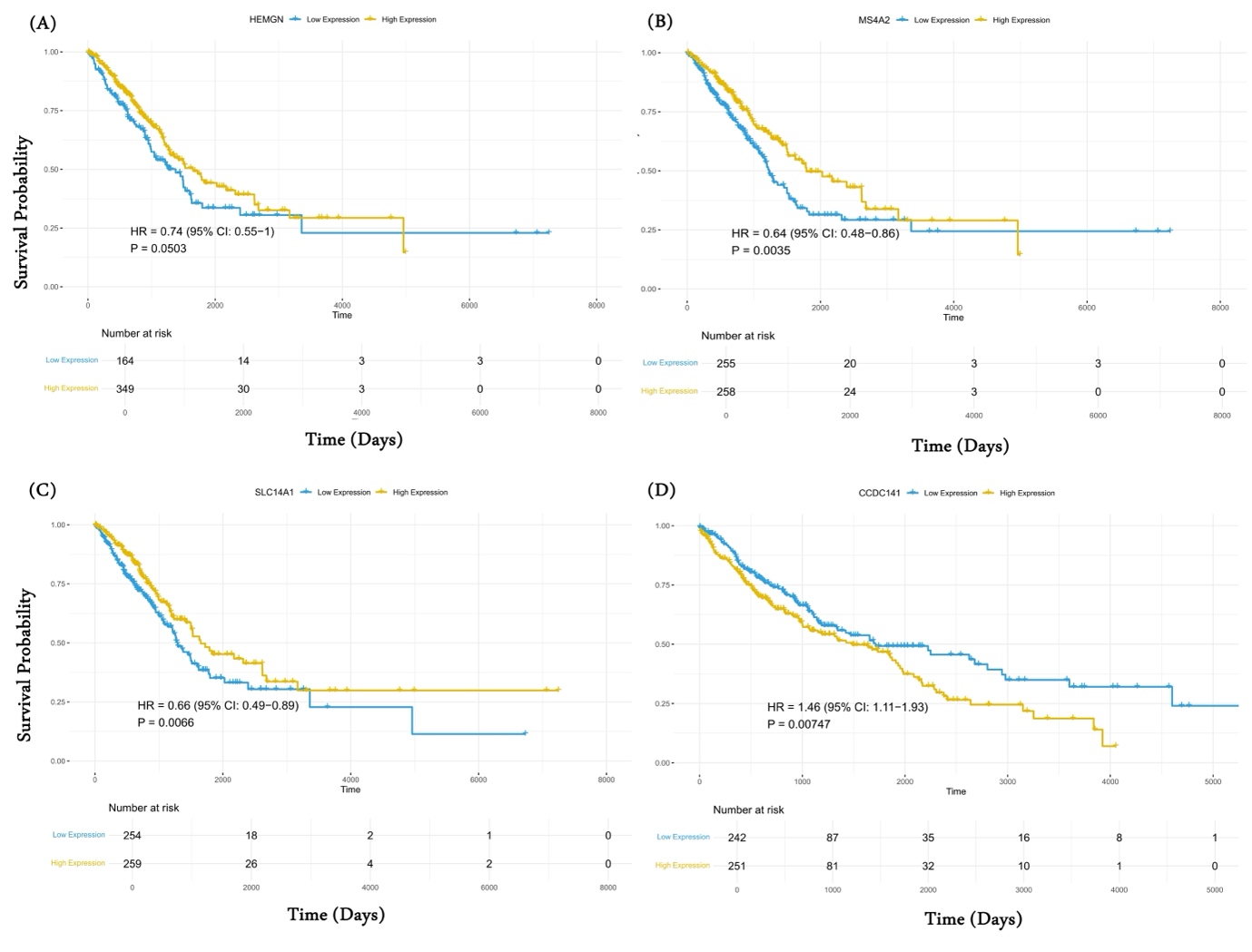


**Supplementary Figure S3. Common Genes are associated with overall survival analysis.** Kaplan Meir (KM) Curve of the 3 significant downregulated genes (A) HEMGN; (B)MS4A2; (C) SLC14A1 in TCGA-LUAD cohort and (D) CCDC141 in TCGA-LUSC cohort; obtained after the Cox regression analysis. For each gene, patients were stratified into high (50%) and low (50%) category based on median expression.

**
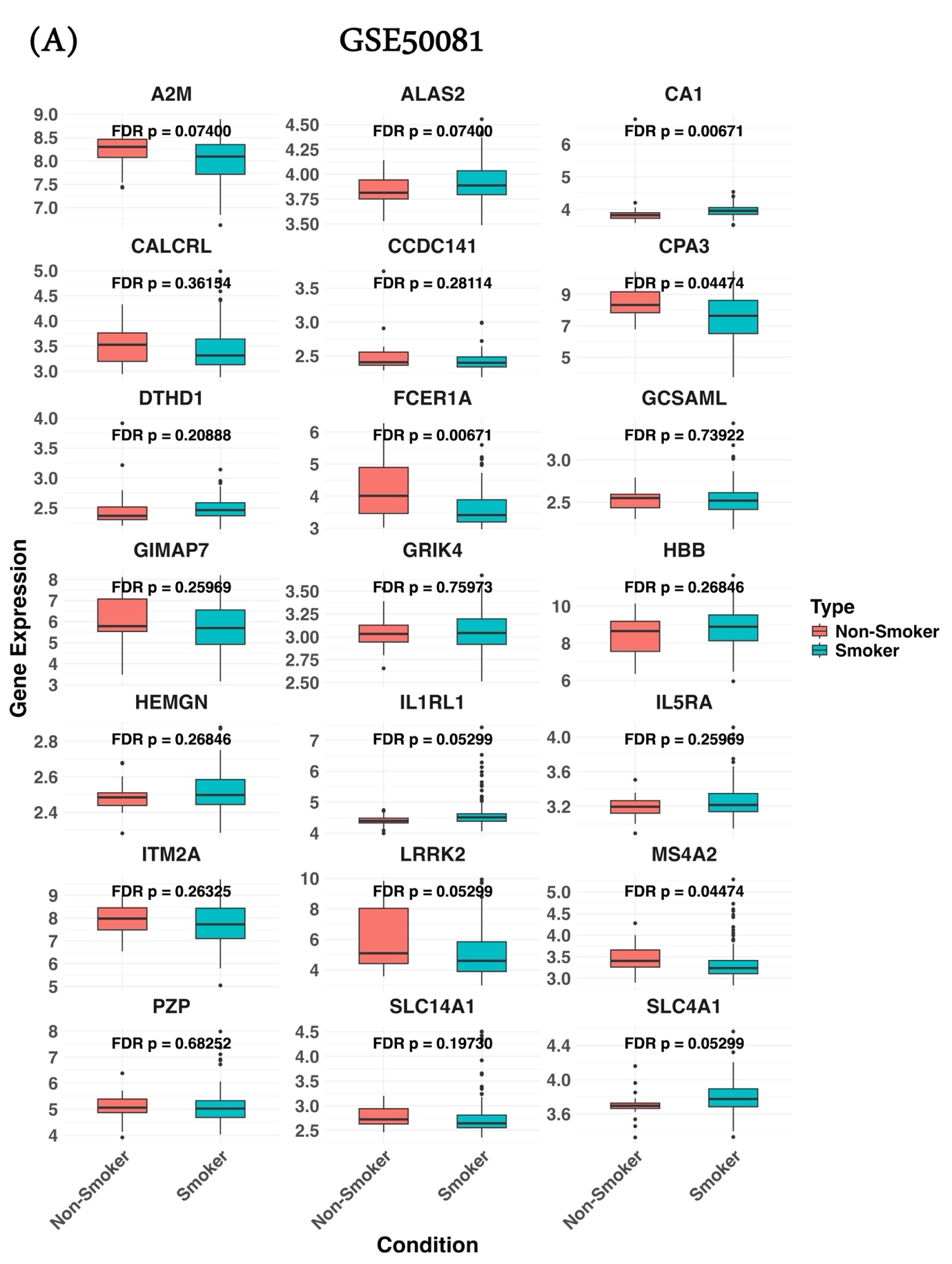
**

**
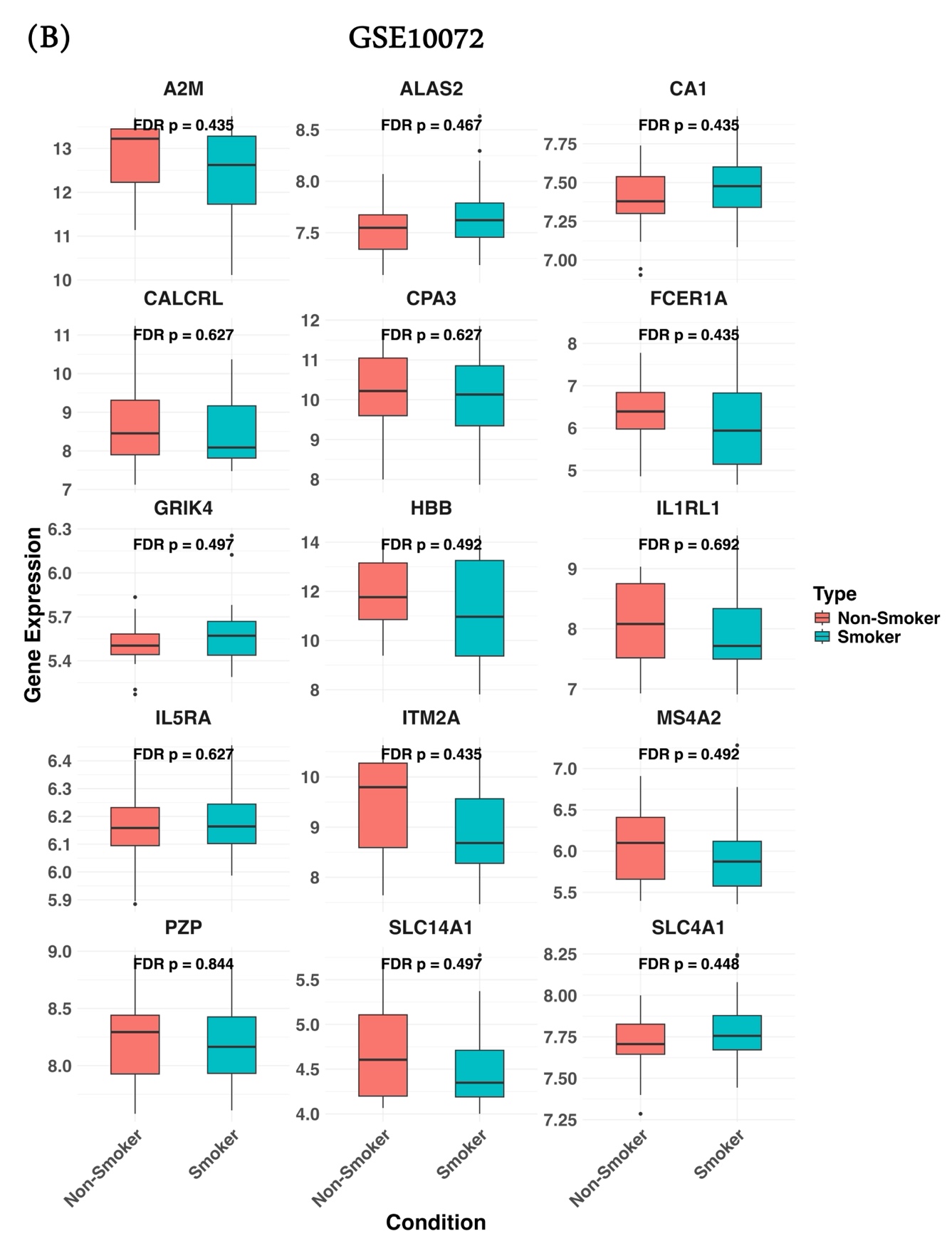
**

**Supplementary Figure S4. Gene Expression Comparison.** Gene expression comparison of common downregulated genes in the lung cancer patients classified as smoker and non-smoker in the dataset (A) GSE50081 and (B) GSE10072. ML model performance was computed in the form of ROC curves and gene Expression comparison was made in the form of boxplot and Wilcoxon test was performed as measure of statistical significance.
